# 312. Performance and validation of an adaptable multiplex assay for detection of serologic response to SARS-CoV-2 infection or vaccination.

**DOI:** 10.1093/ofid/ofac492.390

**Published:** 2022-12-15

**Authors:** Grace Kenny, Sophie R O’Reilly, Riya Negi, Alejandro Garcia-Leon, Dana Alalwan, Colette Marie Gaillard, Gurvin Saini, Rosanna Inzitari, Eoin Feeney, Obada Yousif, Aoife Cotter, Eoghan de Barra, Corinna Sadlier, Fiona Crispie, Peter Doran, Virginie Gautier, Patrick Mallon, M B BCh

**Affiliations:** Centre for Experimental Pathogen Host Research (CEPHR), University College Dublin, Belfield, Dublin 4, Ireland Department of Infectious Diseases, St Vincent’s University Hospital, Elm Park, Dublin 4, Ireland, Dublin, Dublin, Ireland; Centre for Experimental Pathogen Host Research (CEPHR), University College Dublin, Dublin, Dublin, Ireland; Centre for Experimental Pathogen Host Research (CEPHR), University College Dublin, Belfield, Dublin 4, Ireland, Dublin, Dublin, Ireland; Centre for Experimental Pathogen Host Research (CEPHR), University College Dublin, Belfield, Dublin 4, Ireland, Dublin, Dublin, Ireland; Centre for Experimental Pathogen Host Research, University College Dublin, Belfield, Dublin, Ireland; Centre for Experimental Pathogen Host Research (CEPHR), University College Dublin, Belfield, Dublin 4, Ireland, Dublin, Dublin, Ireland; Centre for Experimental Pathogen Host Research (CEPHR), University College Dublin, Belfield, Dublin 4, Ireland, Dublin, Dublin, Ireland; School of Medicine, University College Dublin, Belfield, Dublin, Ireland; Department of St Vincent's University Hospital and Centre for Experimental Pathogen Host Research University College Dublin, Belfield, Dublin, Ireland; Endocrinology Department, Wexford General Hospital, Carricklawn, Wexford, Ireland, Wexford, Wexford, Ireland; Centre for Experimental Pathogen Host Research (CEPHR), University College Dublin, Belfield, Dublin 4, Ireland Department of Infectious Diseases, Mater Misericordiae University Hospital, Eccles St, Dublin 7, Ireland, Dublin, Dublin, Ireland; Department of Infectious Diseases, Beaumont Hospital, Beaumont, Dublin 9, Ireland Department of International Health and Tropical Medicine, Royal College of Surgeons in Ireland, Dublin, Ireland, Dublin, Dublin, Ireland; Department of Infectious Diseases Cork University Hospital, Cork, Cork, Ireland; Teagasc Food Research Centre, Moorepark, and APC Microbiome Ireland, Cork, Cork, Ireland; 3School of Medicine, University College Dublin, Belfield, Dublin 4, Ireland, Dublin, Dublin, Ireland; Centre for Experimental Pathogen Host Research (CEPHR), University College Dublin, Belfield, Dublin 4, Ireland, Dublin, Dublin, Ireland; University College Dublin, Dublin, Dublin, Ireland

## Abstract

**Background:**

A wide array of assays to detect the serologic response to SARS-CoV-2 have been developed since the emergence of the pandemic. The majority of these are either qualitative or semi-quantitative, detect antibodies against one antigenic target, and are not adaptable to new antigens.

**Methods:**

We developed a new, multiplex immunoassay to detect antibodies against the receptor binding domain, S1 and S2 spike subunits and nucleocapsid (N) antigens of SARS-CoV-2 (the CEPHR SARS-CoV-2 Serology Assay). This assay uses electrochemiluminescence technology which allows for a broad dynamic range, and a linker format which allows for the addition of new antigenic targets. We tested this assay on a series of biobanked samples and validated its performance against the Abbott SARS-CoV-2 IgG and Abbott SARS-CoV-2 IgG II assays, and the MesoScale Diagnostics V-PLEX SARS-CoV-2 Panel 2 Kit.

**Results:**

Participant demographics are shown in Table 1. The mean (standard deviation (SD)) intra-assay (within plate) coefficient of variation (CV) of 80 plasma samples run on the same plate was 3.9% (2.9) for N, 3.8% (6.2) for RBD, 3.8% (5.9) for S1 and 3.9% (5.3) for S2. The mean (SD) inter-assay CV derived from 5 samples run across 3 days by two different operators was 11% (6.5) for N, 13% (5.7) for RBD, 14% (8.9) for S1 and 13% (5.1) for S2. In the convalescent group (n=193), overall sensitivity for each assay was; RBD 82% (95% CI 76-87), S1 86% (81-91%), S2 88% (83 – 92%) and N 72% (64 – 78%). Sensitivity improved when analysis included only individuals who were sampled more than 14 days from onset of symptoms (n=166), RBD 87% (81 – 95%), S1 91% (85 – 95%), S2 91% (85 – 95%) but not for the N-target (73% (66-80%)). In vaccinated individuals (n = 58), 100% (94-100%) had both detectable RBD and S1 antibodies. Overall specificity was 96% (87-99%) for RBD, 90% (78-97%) for S1, 94% (84-99%) for S2 and 90% (78-97%) for N. There was excellent correlation between the Abbott IgG II and both CEPHR anti-RBD IgG (rho 0.91) and CEPHR anti-S1 IgG (rho 0.9, both p < 0.001, Figure 1.) and the V-PLEX full spike and both CEPHR RBD IgG (rho 0.83) and S1 IgG (rho 0.82, both p < 0.001, Figure 4).

**Table 1:**
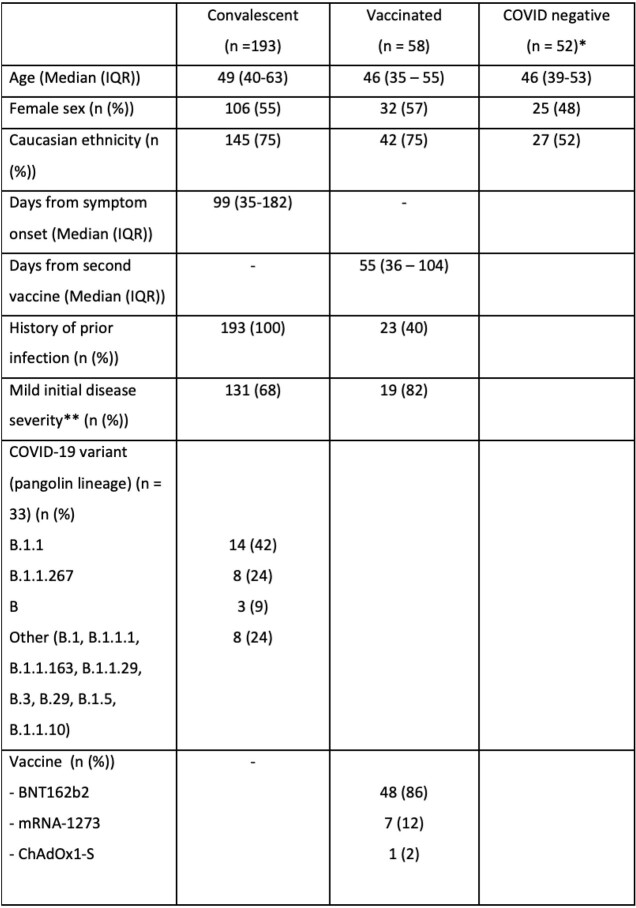
Participant demographics

Correlation between CEPHR RBD, Abbott SARS-CoV-2 IgG II anti spike assay and V-PLEX Spike assay.

**Figure:**
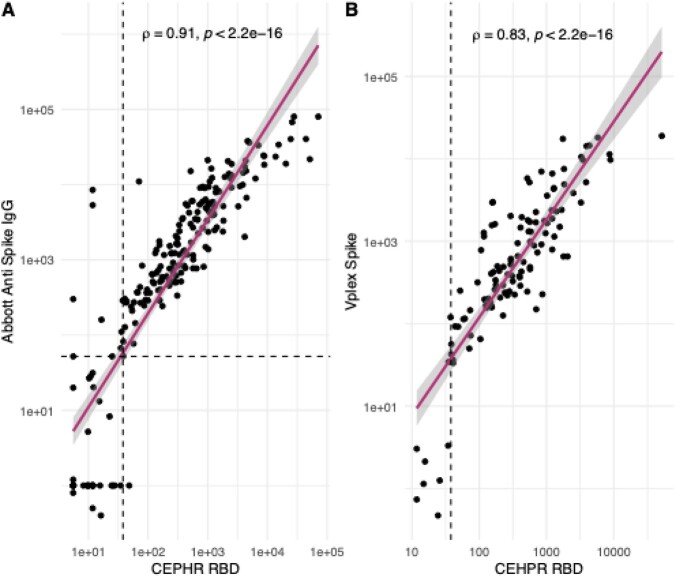

Vertical dashed line represents CEPHR RBD positivity threshold, horizontal dashed line indicates Abbott positivity threshold. B: Correlation between CEPHR RBD and MSD V-PLEX Spike IgG. Vertical dashed line represents CEPHR RBD positivity threshold, no positivity threshold provided by MSD.

**Conclusion:**

The CEPHR SARS-CoV-2 Serology Assay is a robust, customisable, multiplex serologic assay for the detection of several different IgG specific to SARS-CoV-2.

**Disclosures:**

**All Authors**: No reported disclosures.

